# Medical Therapy of Patients Contaminated with Radioactive Cesium or Iodine

**DOI:** 10.3390/biom9120856

**Published:** 2019-12-11

**Authors:** Jan Aaseth, Valeria Marina Nurchi, Ole Andersen

**Affiliations:** 1 Research Department, Innlandet Hospital Trust, 2381 Brumunddal, Norway; 2 IM Sechenov First Moscow State Medical University (Sechenov University), 119146 Moscow, Russia; 3 Department of Life and Environmental Sciences, University of Cagliari, Cittadella Universitaria, 09042 Monserrato-Cagliari, Italy; nurchi@unica.it; 4 Department of Science, Systems and Models, Roskilde University, 4000 Roskilde, Denmark; oa@ruc.dk

**Keywords:** cesium, radioactive terrorism, radiation dosage, chelation therapy, iodine, strontium, Prussian blue

## Abstract

Follow-up studies after the Chernobyl and Fukushima accidents have shown that ^137^Cs and ^131^I made up the major amount of harmful contaminants in the atmospheric dispersion and fallout. Other potential sources for such radionuclide exposure may be terrorist attacks, e.g., via contamination of drinking water reservoirs. A primary purpose of radionuclide mobilization is to minimize the radiation dose. Rapid initiation of treatment of poisoned patients is imperative after a contaminating event. Internal contamination with radioactive material can expose patients to prolonged radiation, thus leading to short- and long-term clinical consequences. After the patient’s emergency conditions are addressed, the treating physicians and assisting experts should assess the amount of radioactive material that has been internalized. This evaluation should include estimation of the radiation dose that is delivered and the specific radionuclides inside the body. These complex assessments warrant the reliance on a multidisciplinary approach that incorporates regional experts in radiation medicine and emergencies. Regional hospitals should have elaborated strategies for the handling of radiation emergencies. If radioactive cesium is a significant pollutant, Prussian blue is the approved antidote for internal detoxification. Upon risks of radioiodine exposure, prophylactic or immediate treatment with potassium iodide tablets is recommended. Chelators developed from calcium salts have been studied for gastrointestinal trapping and enhanced mobilization after strontium exposure.

## 1. Introduction

Although radioactive elements may occur naturally in the environment, most of the harmful radionuclides are of anthropogenic origin, released from military, industrial or medical processes [1]. Nuclear reactor accidents can lead can lead to workers’ exposure. However, in recent years great concerns have been raised regarding contamination of the general environment. Reports after the Chernobyl and Fukushima accidents revealed that the radioisotopes ^137^Cs and ^131^I made up the major amount of harmful contaminants in the air dispersion and fallout [2]. Today, terrorist attacks may represent another potential source for radionuclide exposure of larger populations, e.g., through contamination of drinking water. A nuclear explosion by terrorists is a worst-case scenario. Also, inadvertent exposure due to radiation from radioactive sources used in a medicine or industry may happen. Human uptake of radionuclides may occur via contaminated food or dust. A primary purpose of emergency therapy mobilization is to minimize the radiation dose of contaminated individuals [3,4,5]. Nuclear incidents occur infrequently and some occur very rarely. However, when they do occur, they cause a considerable amount of fear and concern in the public and among emergency physicians and assistant staff, which is partly due to suboptimal healthcare knowledge among the staff about evaluation and care of the victims [6].

The aim of the present review is to give an update of therapeutic and prophylactic measures for obtaining radionuclide protection and mobilization of cesium and iodine from exposed individuals after accidents. Problems related to strontium mobilization are only briefly commented on.

## 2. General Principles in Therapy of Contaminated Cases

Emergency department staff members who initially evaluate patients contaminated with radioactive material should work closely with a nuclear medicine expert. This expert should be an integral part of the team receiving exposed patients in a dedicated area of the emergency department. The trained staff in CBRN medicine, which includes medical handling of patients after Chemical, Biological, Radiological and Nuclear accidents, in case of nuclear accidents should work in close cooperation with the expert in nuclear medicine, who should take part in the important administrative steps, which can be summarized as follows [5]:

(a) Define a radiation work area or room;
(b) Define personal protective equipment;
(c) Introduce individual dosimeters to staff members;
(d) Monitor contamination on patients.

In an emergency situation, a limit for the radiation dose to the emergency staff is not set when they perform the initial life-saving activities. However, a cumulative dose limit (0.5 Gy) has been suggested as a decision point for the team leader when considering how to proceed after an initial time period of about 60 min [5].

Contaminated clothes of a patient are removed carefully and stored away. Radiation monitoring may then be necessary to locate any skin or wound contamination. Decontamination of the skin with water and soap, and of wounds with physiological saline will help to decrease the risk of additional intake of radioactive material into the body and to decrease the total radiation exposure. Cleaning of wounds should be done with care. All water used for the external cleaning is collected and treated as contaminated material. After the external decontamination, the radiation monitoring is repeated to ensure that external purification can be discontinued.

As soon as a patient is externally decontaminated, a clinician specialized in nuclear and/or CBRN medicine needs to assess whether the patient is internally contaminated with radioactive material. The goal of assessing internal contamination is to evaluate whether the patient has taken up hazardous amounts of the material to deliver a significant dose of radiation to the body or to specific organs, like the thyroid gland in the case of radioactive iodine. The clinical concern includes also long-term consequences like cancer. Cancer may result from cumulative radiation dose over time. The clinician should consider therapies that facilitate the removal of the radioactive material.

The presence as regards type and amount of radioactive material inside the body can be assessed directly when the patient remains in the radiation area, by using an external radiation detector such as a hand-held radiation Geiger counter, provided that the element emits gamma rays. Nuclear medicine departments of regional hospitals potentially have equipment that can be adapted for the diagnosis of internal contamination and internal dose assessment, for example, thyroid scanners or gamma cameras otherwise used for SPECT imaging, but these more accurate determinations may require that the externally decontaminated patient is carefully transported to the specialized department.

The management of internal contamination includes, in addition to supportive care, measures to decrease the radiation dose that is delivered to body organs and tissues. This measurement can be achieved by decreasing the absorption of the radioactive element from the gastrointestinal tract or by enhancing excretion in urine or feces, or by blocking the incorporation into specific organs. The approaches recommended for mobilization of radioactive species of cesium and iodine, which dominate in the environment after nuclear reactor accidents, are discussed below.

## 3. Radioactive Cesium Contamination

**Figure 1:**
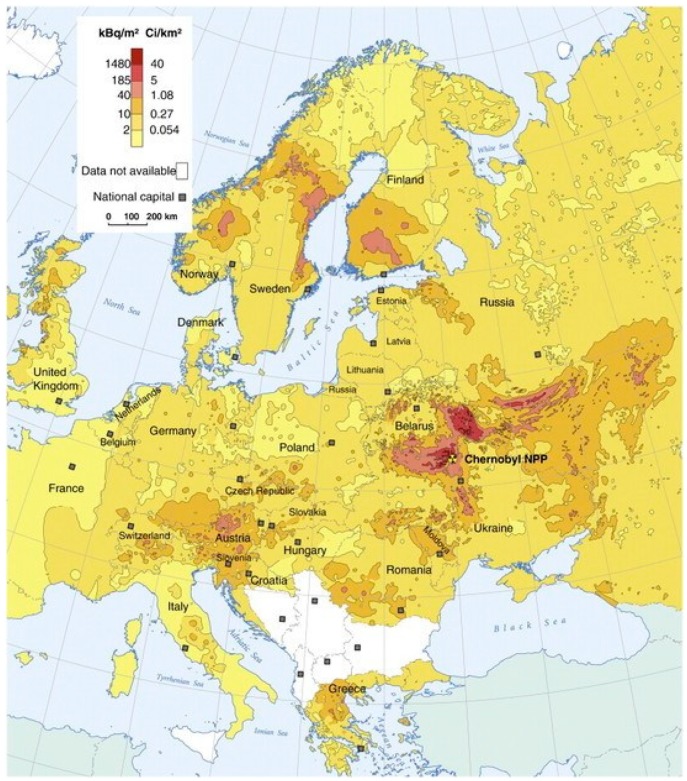
Radiocesium fallout in Europe after the Chernobyl accident (adapted from Matisoff and Whiting, 2012 [7]).

Cesium (Cs, atomic number 55) in Group 1 of the periodic table of elements has standard atomic weight 132.9. It occurs as the monovalent cation Cs^+^ in chemical compounds. Chemically, Cs has similarities with other elements in Group 1, such as sodium and potassium. It is a soft, malleable, silvery white metal with melting point as low as 28.4 °C. Cesium has 40 known isotopes, of which only ^133^Cs is stable. After nuclear reactor accidents, the ^137^Cs isotope is considered a most dangerous pollutant in the fallouts. The ^137^Cs isotope has physical half-life of about 30 years, whereas ^134^Cs has a shorter half-life of about 2 years. Fission of uranium and plutonium in nuclear reactors and nuclear weapons due to neutron absorption creates numerous fission products, including ^134^Cs and ^137^Cs. The first one of these two isotopes, ^134^Cs, decays with both β and γ emission. The second one, ^137^Cs, also decays with β and γ emissions, to the metastable ^137m^Ba. After the Chernobyl and Fukushima accidents, the ^137^Cs isotope was among the most important environmental pollutants [2] (Figure 1).

However, ^137^Cs has also a wide range of practical uses. In medicine, it has been used in cancer radiation therapy. In industry it has been used in moisture-density gauges and leveling gauges.

Absorbed ^137^Cs is uniformly distributed in soft tissues, resembling the physiological distribution of potassium, with slightly higher levels in muscles and lower levels in bone and fat. It is excreted in urine, an initial fraction with a biological halftime of only 3 days, whereas the major part of absorbed Cs is excreted with a halftime of almost 3 months [8].

### Radioactive Cesium Antidotes

According to the HSAB theory of Pearson [9,10], Cs^+^ as well as Sr^2+^ belong to the group of so-called “hard metals” with high affinity to ligands with “hard electron donor groups”, which are chemical groups containing oxygen and also to some extent nitrogen atoms. Prussian blue, also known as Berlin blue, with appropriate donor groups is anticipated to be a therapeutically useful antidote according to the theory, provided that it is administered early after intake, to trap the metal in the gut and thus also from blood due to the enterohepatic circulation of the element. Nielsen et al. [11] studied effects of two Prussian blue derivatives on intestinal absorption of ^134^Cs in two male volunteers. Their results indicated that administration of Prussian blue (0.5 g) simultaneously with the test meal decreased ^134^Cs uptake to about 50%, and that daily administration of 0.5 g × 3 decreased the elimination half time of previously absorbed ^134^Cs from about 100 to about 50 days. Melo et al. [12] investigated the effect of age on the decontamination effect of Prussian blue using male beagle dogs injected with ^137^Cs. The studied dogs were either immature (about 5 months), young adults (about 2.5 years), or aged (13.5 years). The researchers observed that ^137^Cs excretion rates decreased with increasing age. Thus, the reductions in whole-body levels from Prussian blue chelation were 51% in immature, 31% in young adults and 38% in aged dogs. Prussian blue changed the ratio of fecal to urinary ^137^Cs excretion from 0.8 in untreated dogs to 2.2 in treated animals. The hepatic ^137^Cs levels were reduced in all chelated dogs. Indirectly, Prussian blue will also mobilize ^137^Cs from the bloodstream by intervening with its enterohepatic circulation [13].

Le Gall et al. [14] compared the ^137^Cs-mobilizing effect of apple-pectin and Prussian blue in rats intravenously injected with 5 kBq of ^137^Cs. The mobilizing agents were given in drinking water at a dose of 400 mg/kg per day during an 11-day period, starting immediately after the Cs injection. Prussian blue increased the fecal Cs excretion by a factor of five, whereas Cs elimination after the apple-pectin treatment was not increased compared with untreated rats.

After the Goiania accident in Brazil in 1987, where a radiotherapy source containing an estimated 50.9 TBq of ^137^Cs was stolen from an abandoned hospital and broken open, Brandao-Mello et al. [15] and Farina et al. [16] described promising results of early oral treatment with Prussian blue of some of the exposed patients. After this accident, 249 cases out of 112,000 people examined for contamination had significant internal or external radioactive contamination [17]. Internal contamination was verified by the analysis of urine and fecal samples and in a whole-body counter. The first report described 14 patients who developed severe bone marrow depression with neutropenia and thrombocytopenia. Eight of these received intravenous granulocyte-macrophage colony-stimulating factor; none had bone marrow transplantation. Four died from hemorrhage and infections. Patients with extensive internal contamination were chelated by Prussian blue at doses from 1.5 to 10 g/day. Other measures to increase ^137^Cs elimination included diuretics combined with water or electrolyte treatment. Mobilization of ^137^Cs in patients less severely affected was also described. Oral Prussian blue was administered to 46 patients, and 17 patients received oral diuretics. The therapeutic regimen was described as successful.

Based on experimental and clinical observations, the clinically recommended ^137^Cs antidote is Prussian blue given orally early after poisoning [13]. While this drug may be lifesaving after oral exposure, its efficacy after inhalation of ^137^Cs-contaminated dust is questionable. At present, no chelating drug is available as nebulizer for mobilization of pulmonary-deposited radioactive substances. However, the US Food and Drug Administration (FDA) has approved orally given Prussian blue as a drug for people exposed to radiation contamination (cesium and thallium). The drug is available as Radiogardase capsules. In addition to oral Prussian blue treatment, acute cases may require intravenous infusion with appropriate fluids and electrolytes. Adequate clinical chemistry monitoring, including measurements of blood cell counts, is routinely done. Maintenance of adequate potassium levels in blood is important. Development of bone marrow depression with leukopenia may require treatment with a stimulating factor, e.g., Neupogen. In severe cases of pancytopenia, bone marrow transplantation may be necessary.

## 4. Radioactive Strontium Contamination

Strontium (Sr), atomic number 38, in Group 2 of the periodic table of elements, standard atomic weight 226, density 5.5 g/cm^3^, is a soft silver-white chemically highly reactive metal. Chemically, Sr^2+^ ions have similarities with Ca^2+^ and Mg^2+^. Strontium has several known isotopes, of which ^89^Sr occurs as pollutant around nuclear facilities, and ^90^Sr is formed as a byproduct during fission of plutonium and uranium in nuclear reactors and nuclear weapons. Large amounts of ^90^Sr were dispersed worldwide during nuclear weapons tests in the 1950s and 1960s. ^90^Sr decays with β emission and a half-life of about 29 years. It has been used in the therapy of bone metastases. The intestinal uptake of Sr^2+^ is about 30%. Absorbed Sr^2+^ is mainly deposited in bone with a very long biological half-time, and thus radioactive strontium increases the risk of bone cancer and leukemia.

### Radioactive Strontium Antidotes

Jagtap et al. [18] compared the ^85^Sr mobilizing effects several calcium salts, including Ca gluconate (CaG), Ca lactate (CaL), Ca carbonate (CaC) and Ca phosphate (CaP), with that of Ca alginate (CaA), the latter often advised for Sr^2+^ mobilization. Rats given ^85^Sr i.p. or orally were treated with one of the Ca salts 2 h after ^85^Sr administration and thereafter once daily. The Ca salts were administered orally except for CaG, which was administered i.p. in the i.p. Sr group. The diet of experimental groups was supplemented with the respective Ca salts to 2% elemental Ca. The therapy with Ca salts significantly reduced the whole-body retention of ^85^Sr to about half of the retention in controls, as measured after two weeks. These results indicate that any commonly used Ca salts could replace Ca alginate for reducing ^85^Sr body burden.

The clinical use of commercial Ca alginate for internal ^85^Sr decontamination is limited by its low solubility. Levitskaia et al. [19] (2010) increased the solubility of alginate by using a low molecular weight polyethylene glycol/sodium bicarbonate buffer. Oral administration of this alginate solution removed internal ^85^Sr in rats significantly. The present clinical recommendation for strontium decorporation is early initiated long-term treatment with an oral calcium salt. Adjuvant long-term dietary therapy with alginates may be used. In severe cases of radiation sickness characterized, inter alia, by pancytopenia, bone marrow transplantation may be necessary.

## 5. Radioiodine Contamination

Iodine (I) has atomic number 53 and atomic weight 127. It is well known that iodine is important in nutrition. Iodine’s relatively high atomic number, low toxicity and ease of attachment to organic compounds have made iodine radioisotopes, such as ^131^I, a part of many contrast materials in modern radiology and nuclear medicine. Iodine has only one stable isotope, ^127^I, with 74 neutrons.

Iodine is found on Earth mainly as the water-soluble iodide. Otherwise, free iodine occurs mainly as a di-atomic molecule I_2_. Iodine is the heaviest known essential element to be used by life in vital biological functions. It is required by higher animals as a component of thyroid hormones. This element is rare in many soils, leading to many deficiency problems in inland animals and inland human populations. Like nonradioactive iodine, the radioisotope ^131^I is taken up and concentrates in the thyroid.

**Figure 2:**
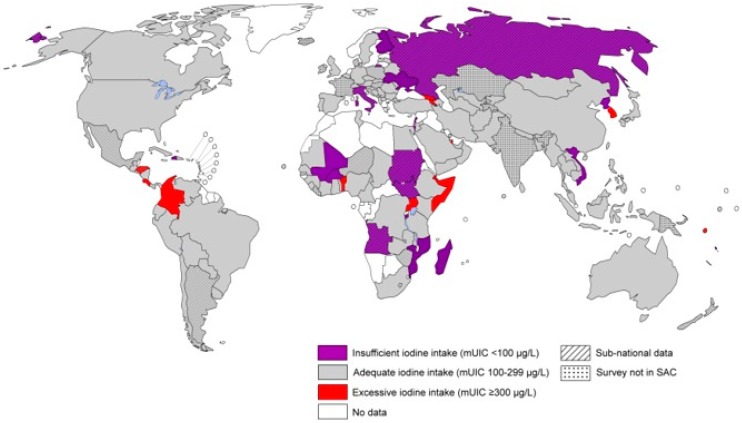
Global map illustrating regions with deficient, adequate or excessive intakes of iodine in the populations. Populations with deficient or low baseline iodine intakes will be particularly susceptible for radioiodine uptake in the thyroid. (Illustration adapted from “Iodine Global Network” Newsroom 2019 [20]).

Radioactive ^131^I has a decay half-life of about eight days. It decays with both β and γ emission. Radioactive ^131^I is used industrially in association with nuclear energy, and plays a major role as a radioactive isotope present in nuclear fission products. This is because ^131^I is a major uranium and plutonium fission product, comprising nearly 3% of the total products. It was a significant contributor to the health hazards from the Chernobyl and Fukushima accidents [2]. Populations with suboptimal or deficient iodine intakes will have increased risk of radioiodine accumulation in the thyroid after such disasters (Figure 2).

The same isotope is also used in medical diagnostics and in treatment procedures. Due to its β decay, ^131^I may cause mutations intracellularly in tissue that it penetrates. High doses of the isotope have been considered less dangerous than low doses, because high doses tend to kill tissue cells rather than acting carcinogenic. Most studies of very high-dosed ^131^I-treatment of Grave’s disease have failed to detect any increase in post-therapeutic thyroid cancer [21]. Medically, ^131^I is now less used in low doses to children but increasingly used in large or maximal treatment doses in adults, as a way of eradicating malignant cells.

In nuclear medicine, ^131^I can be used as an imaging source since about 10% of its radiation dose is via γ radiation. However, since the other 90% of its emission is tissue-damaging β radiation, other emitters such as ^99m^Tc are increasingly used in nuclear radiology today. A less damaging radioisotope of iodine, ^123^I, is preferred when an iodine isotope is required.

Relatively small incidental doses of ^131^I are considered to be the major cause of increased thyroid cancers after accidental nuclear contamination [22,23]. After the Chernobyl accident, the frequency of thyroid cancers in children and in adults increased in regions of Ukraine, Belarus and Russia [24,25,26].

Per capita doses in regions of United States resulting from all exposure routes from all atmospheric nuclear tests conducted at the Nevada Test center in the period 1950–1962 were rather low. However, it has been estimated that nuclear fallout might have led to approximately 11,000 excess deaths, most caused by thyroid cancer linked to exposure to ^131^I [27].

The risk of thyroid cancer appears to diminish with increasing age at time of exposure. Most risk estimates are based on studies in which radiation exposures occurred in children or teenagers. When adults are exposed, it has been difficult to detect a statistically significant difference in the rates of thyroid disease compared to nonexposed groups [28].

In March 2011, the Massachusetts Department of Public Health reported that ^131^I was detected in very low concentrations in rainwater from samples collected in Massachusetts, USA, and that this likely originated from the Fukushima power plant [29]. Farmers near the plant dumped raw milk. Testing in the United States found 0.8 picocuries per liter of ^131^I in a milk sample, these radiation levels being 5000 times lower than the FDA’s intervention level and thus not hazardous at all [30].

### Radioiodine Antidotes

A common treatment method for preventing ^131^I exposure is by saturating the thyroid with regular, nonradioactive ^127^I, as an iodide or iodate salt. When the gland is saturated with nonradioactive iodide, the thyroid will absorb minimal amounts of the radioactive ^131^I, thereby avoiding the adverse effects from radioiodine. The most common method of preventive treatment is to give potassium iodide to those at risk. The dosage for adults is 130 mg potassium iodide per day, given in one dose or divided into portions of 65 mg twice a day. This is equivalent to 100 mg of iodine. However, the preventive ingestion of iodide is not without its dangers. There is reason for caution about taking potassium iodide or iodine supplements if not necessary, as these doses can disclose or worsen hyperthyroidism and hypothyroidism, and ultimately cause permanent thyroid disease. The preventive iodide doses can also cause gastrointestinal disturbances and allergic reactions. Thus, potassium iodide is not recommended for those who have had an allergic reaction to iodine, and people with severe dermatitis or vasculitis that are linked to a risk of iodine sensitivity.

Administration of perchlorate, a known goitrogen, has also been shown to reduce radioiodine uptake. Perchlorate ions competitively inhibit the process by which iodide is actively deposited into thyroid follicular cells. Although perchlorate can disrupt the thyroid function, this substance remains very useful as a single dose application to remove radioiodide accumulated in the thyroid.

In the event of a radioiodine release, the ingestion of prophylactic potassium iodide, if available, should take precedence over perchlorate administration and would be the first line of defense in protecting the population from a radioiodine release. However, in the event of a radioiodine fallout and contamination too widespread to be controlled by the stock of iodide, the distribution of perchlorate tablets would be a cheap second line of defense against carcinogenic radioiodine bioaccumulation.

## 6. Concluding Remarks

The main purpose of radionuclide mobilization is to minimize the radiation of severely affected individuals and, in some cases, also remove the chemical insult, in acute cases by measures administered in a dedicated emergency unit. Since some of the important metal radionuclides have very long biological half-times after deposition in bone, liver or kidneys, rapid initiation of mobilization treatment is imperative, to reduce uptake from skin, wounds, lungs or gastrointestinal tract, and to enhance excretion of circulating radionuclide before tissue deposition. The trained staff in CBRN medicine should in nuclear accidents work in close cooperation with the expert in nuclear medicine, who should take part in the important steps in the patient evaluation.

Major strategies in the acute phase include physical cleaning of skin and wounds. In oral exposures with radiocesium, orally administered Prussian blue can reduce systemic absorption. In the chronic phase, long-term systemic or oral chelation can promote renal or biliary/fecal excretion of circulating radionuclides.

Important examples are as follows:

(a) For Cs decorporation, oral Prussian blue is the treatment of choice. In highly contaminated individuals, such as those in the Goiania accident, this treatment resulted in reduction of whole-body radiation by a factor of about two. Prussian blue insoluble capsules (Radiogardase, Heyl Co, Germany) are approved by the FDA for the treatment of internal contamination with radioactive cesium or thallium. The drug binds cesium in the intestinal tract, thereby also interrupting the enterohepatic circulation. The resulting excreted complex reduces the effective time of residence of the radioactive element inside the body. The dose in adults is 3 g given orally every 8 h for at least 30 days. The duration of therapy is guided by the amount of radioactive cesium that is removed.

(b) For strontium, the present treatment is early initiation and long-term treatment with oral calcium salts.

(c) Upon exposure to radioiodine, potassium iodide tablets in appropriate doses are the recommended preventive drug. The US Food and Drug Administration (FDA) as well as the European Commission for Radiation Protection including the Norwegian Radiation and Nuclear Safety Authority have approved the use and preventive distribution of potassium iodide [31,32]. This drug (KI) is used to saturate the thyroid gland with stable iodine and thus prevent additional subsequent uptake of radioactive iodine. To be efficacious, KI should be taken before or shortly after the internalization of radioactive iodine. The dose of KI depends on the age of the patient. The FDA-approved doses of potassium iodide are as follows: infants less than 1 month old, 16 mg; children 1 month to 3 years, 32 mg; children 3 to 18 years, 65 mg; adults 130, mg [33]. Children younger than 18 years of age and pregnant women (because of the fetus they are carrying) are more vulnerable to the effects of radioactive iodine and should be prioritized to receiving this therapy when indicated.

Unfortunately, KI supplements were not distributed to populations living near to the Chernobyl nuclear power plant after the disaster [25]. On the other hand, the recent accident in Fukushima highlighted the common misconception that KI is a universal antiradiation pill. Many people took potassium iodide tablets without proper indication. These actions can cause harm rather than good to the health of patients [34].
